# Purification and characterization of antioxidant peptides from cooked eggs using a dynamic in vitro gastrointestinal model in vascular smooth muscle A7r5 cells

**DOI:** 10.1038/s41538-018-0015-7

**Published:** 2018-04-23

**Authors:** Jiapei Wang, Wang Liao, Chamila Nimalaratne, Subhadeep Chakrabarti, Jianping Wu

**Affiliations:** 1 Department of Agricultural, Food and Nutritional Science, University of Alberta, Edmonton, Canada; 2 Cardiovascular Research Centre, University of Alberta, Edmonton, Canada

**Keywords:** Peptides, Peptides

## Abstract

Antioxidant peptides derived from food sources are considered as safer alternatives to commercially available antioxidant drugs. As one of the most abundant protein sources, hen’s egg proteins were extensively used to produce antioxidant peptides by enzymatic hydrolysis. Our previous work indicated that gastrointestinal digestion of cooked eggs significantly increased the antioxidant activity due to hydrolysis of egg proteins. To characterize the responsible antioxidant peptides, cooked eggs were digested in a simulated in vitro model of human gastro-intestinal digestion. Prepared digests were fractionated with FPLC (Fast Protein Liquid Chromatography) and RP-HPLC (Reverse-Phase High-Performance Liquid Chromatography) and the antioxidant activity was determined in A7r5 cells (vascular smooth muscle cell line). Further identification of peptides from peptide fractions with the highest antioxidant activity was carried out using LC-MS/MS. Four peptides derived from ovalbumin, DSTRTQ (48–53), DKLPG (61–65), DVYSF (96–100), and ESKPV (205–209), were identified; of which DKLPG did not show antioxidant activity in cells. Enzyme cleave analysis suggested that these four peptides were likely released from ovalbumin only by pepsin non-specific cleaves. It is postulated that egg consumption may exert protection against oxidative stress on human health due to release of antioxidant peptides during digestion.

## Antioxidant peptides from eggs: more active upon digestion

Chicken eggs are a major source of dietary proteins, and can be used to produce antioxidant peptides, safer alternatives to synthetic antioxidant drugs. Jianping Wu and co-workers at University of Alberta, Canada, looked into how digestion of cooked eggs in the stomach and intestines could affect the antioxidant activity. Using a simulated model of human digestion, they separated the digests and identified the most active antioxidant peptides, likely released from ovalbumin—the main protein in egg white—by pepsin, the essential digestive enzyme in the stomach. These findings show that digestion of cooked eggs breaks down egg proteins to more potent antioxidant peptides, suggesting that consumption of eggs may help protect human body against oxidative stress.

## Introduction

Eggs are a nutritious food commodity and an inexpensive source of high-quality proteins. Egg proteins are widely used in the food and nutraceutical industry due to their unique nutritional and functional properties.^1^ Protein accounts for approximately 10% (w/w) of fresh egg white and about 16% in egg yolk.^2^ Almost all egg lipids exist in yolk, representing two-third of its dry weight. Egg yolk lipids exist as lipoprotein assemblies, including low-density lipoproteins and high-density lipoproteins.^2^ The presence of cholesterol in egg yolk was once a concern of egg consumption; however, a cause–effect relationship between dietary cholesterol intake and coronary heart disease incidence has not been established.^3,4^ On the other hand, numerous research works suggest egg is a rich source of bioactive compounds that egg consumption may be beneficial to human health beyond basic nutritional value.^5–7^ Egg constituents including peptides and proteins, lipids and phospholipids, carotenoids have been reported to exert antimicrobial, antioxidant, antihypertensive, immunomodulatory, and anticancer properties in chemical, cellular, and animal experiments.^8–10^

Peptides derived from egg white proteins were reported to have good antioxidant properties. Two peptides derived from egg white peptic hydrolysates, YAEERYPIL and SALAM, showed oxygen radical absorbance capacity (ORAC) of 3.8 and 2.7 µmol, respectively.^11^ Our previous research suggested that egg yolk is also a rich source of antioxidants including free aromatic amino acids.^12^ Eggs are usually cooked and then consumed. In our previous studies we observed that cooking of eggs reduced the antioxidant activity,^12^ while substantially enhanced when cooked eggs were subjected to simulated gastrointestinal digestion using an advance digestion model (TIM-1 TNO’s intestinal model).^13^ The content of FAA were also increased in digested eggs; however, the increment of FAA was not directly related to the increase of the antioxidant activity, suggesting that apart from the free amino acids, peptides generated as a result of the digestion could be a major contributing factor for the increased antioxidant activity.^13^ Nevertheless, the responsible peptides are yet to be characterized.

Antioxidant activity is most commonly assessed by assays such as DPPH (1,1-diphenyl-2-picryl-hydrazyl) radical scavenging activity, hydroxyl radical scavenging activity, ABTS—(2,2′-azinobis(3-ethylbenzothiazoline-6-sulfonate)) radical anion scavenging activity, oxygen radical scavenging capacity, oxygen radical absorbance capacity-fluorescein (ORAC-FL) assay, FRAP (ferric reducing antioxidant potential), superoxide anion scavenging activity, and lipid peroxidation inhibition activity,^14–17^ but these chemical methods have been criticized for lack of biological relevance.^18–21^ Consequently, biologically relevant cell-based antioxidant assays are considered as a better alternative to evaluate the protective effects of antioxidants against oxidative stressors.^21,22^ Hence, the current study was designed to purify and identify the peptides and to confirm their antioxidant activity in more biologically relevant experimental setting. A7r5 cells is a vascular smooth muscle cell (VSMC) line from rat aorta. In our previous study, A7r5 cells have been used to evaluate the antioxidant activity of food protein-derived bioactive peptides focusing on the potential of the peptides in improving vascular health.^23–25^

Given such a background, objectives of this study were to collect the cooked egg digests for a series of fractionation by liquid column chromatography techniques and antioxidant activity of the fraction was screened based on A7r5 cells. The digested egg samples collected were fractionated using a series of liquid chromatography and screened for antioxidant activity on A7r5 cells (VSMC line). Peptide sequences in the most active fractions were determined using liquid chromatography–tandem mass spectrometry (LC-MS/MS). Antioxidant activity of the identified peptides were synthesized and the activity was verified using A7r5 cells.

## Results and discussions

### Preparation and purification of the whole cooked egg digests

The whole cooked egg was digested with a modified TIM-1 system and the bioaccessible digests were collected by passing through a specially designed membrane to simulate the digestion and absorption process in vivo.^26^ After defatting, the bioaccessible egg digests were desalted by passing through a C_18_ cartridge column to obtain three bound fractions, F1, F2, and F3, which were then subjected to antioxidant activity evaluation.

Antioxidant activity is most commonly assessed by chemical methods such as DPPH (1,1-diphenyl-2-picryl-hydrazyl) radical scavenging activity, hydroxyl radical scavenging activity, ABTS—(2,2′-azinobis(3-ethylbenzothiazoline-6-sulfonate)) radical anion scavenging activity, oxygen radical scavenging capacity, ORAC-FL assay, FRAP (ferric reducing antioxidant potential), superoxide anion scavenging activity, and lipid peroxidation inhibition activity,^14–17^ but was criticized for lack of biological relevance.^18–21^ Consequently, biologically relevant cell-based antioxidant assays are considered as a better alternative to evaluate the protective effects of antioxidants against oxidative stressors.^20,22^

**Fig. 1:**
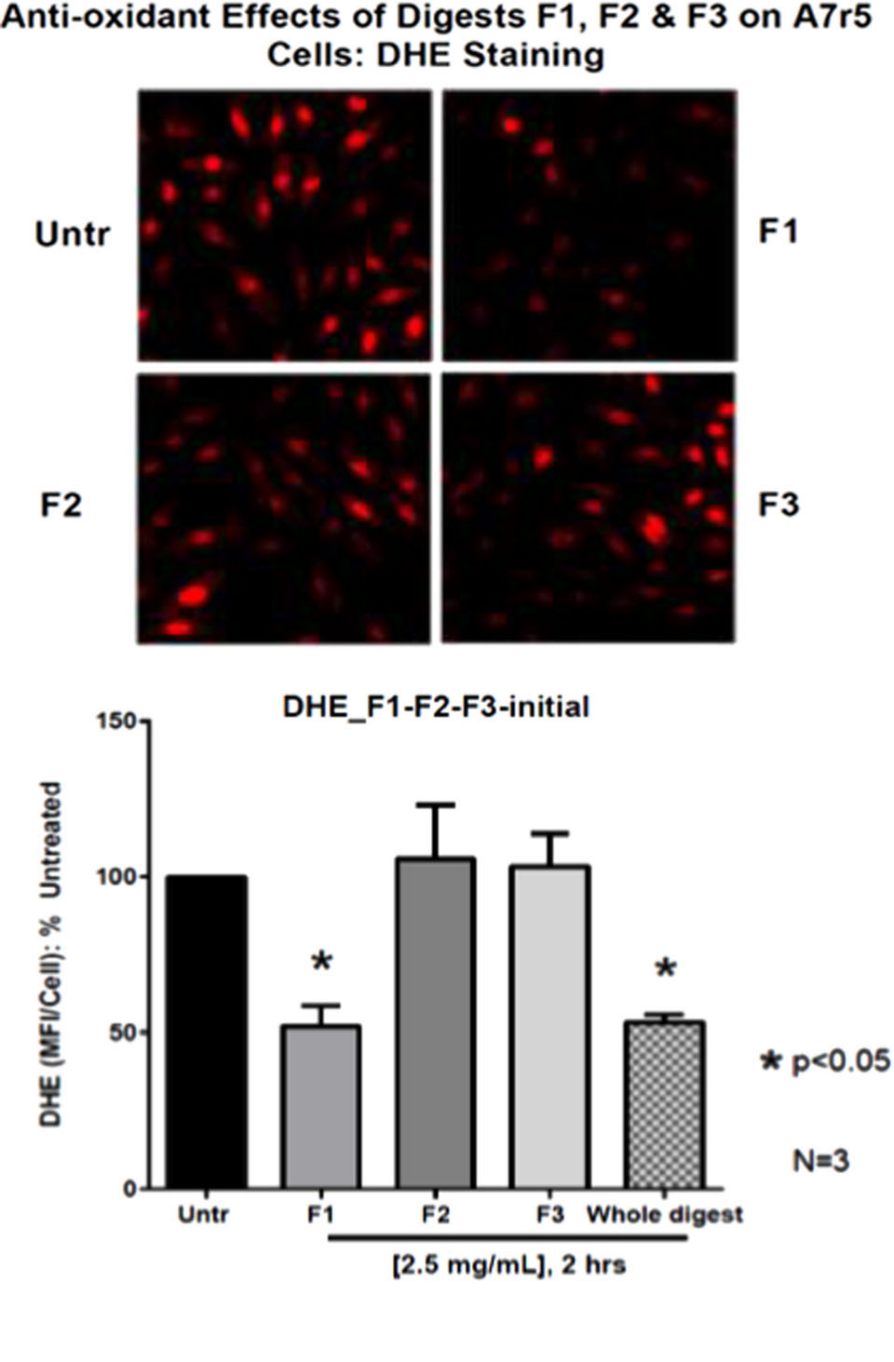
Antioxidant effect of fractions from defatted digests on A7r5 cells. Untr: control; F1, F2, and F3: eluted from a C_18_ cartridge column with 20, 50, and 100% of ACN solution containing 0.1% TFA, subsequently; whole digest: the original digest was simply defatted and desalted. Cultured monolayers of vascular smooth muscle cells (A7r5 cell line) were pretreated with various fractions (with concentration of 2.5 mg/mL) for 2 h and without pretreatment as control. The cells were then washed once and incubated at 37 °C with 10 μM DHE in the DMEM containing 1% FBS for 30 min. Fluorescence intensity from three randomly chosen fields was measured by its mean fluorescence intensity per cell (MFI/cell). Superoxide generation was calculated as fold change in MFI/cell and presented as percentage (%) of the untreated control. Mean ± SEM of three independent experiments are shown. Asterisk indicates *p* < 0.05 compared to the untreated control

**Fig. 2:**
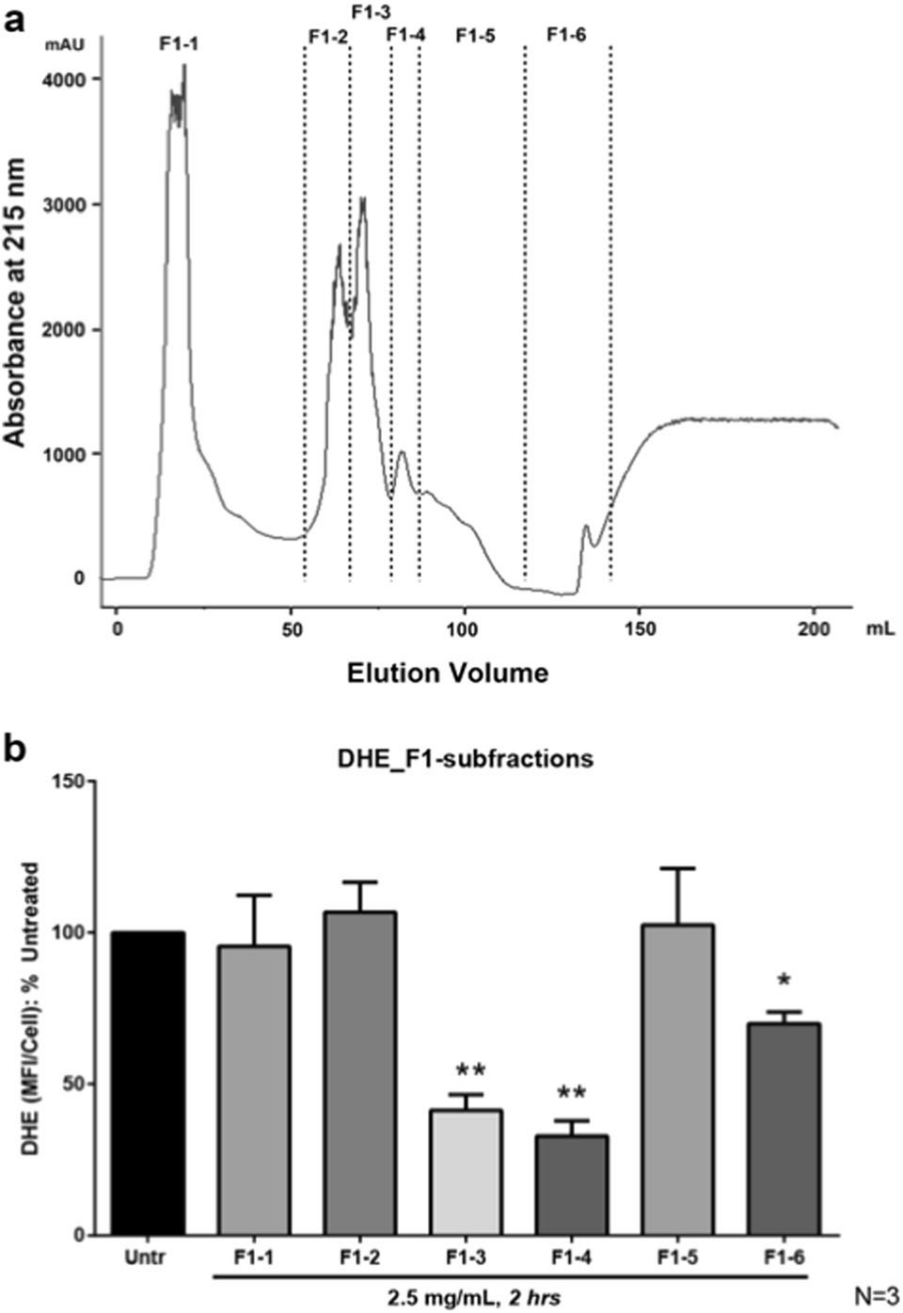
**a** Separation peptide fraction (F1 with the highest antioxidant activity) on a cation exchange (High-Prep 16/10) column coupled with FPLC system and six fractions were obtained; **b** antioxidant effect of peptide fractions from FPLC fractionation on A7r5 cells. Untr: control; F1-1, F1-2, F1-3, F1-4, F1-5, and F1-6: from F1 by FPLC separation. Superoxide generation was expressed as fold change in mean fluorescence intensity per cell (MFI/cell) and presented as percentage (%) of the untreated control. Mean ± SEM of three independent experiments are shown. Asterisk indicates *p* < 0.05 compared to the untreated control

**Fig. 3:**
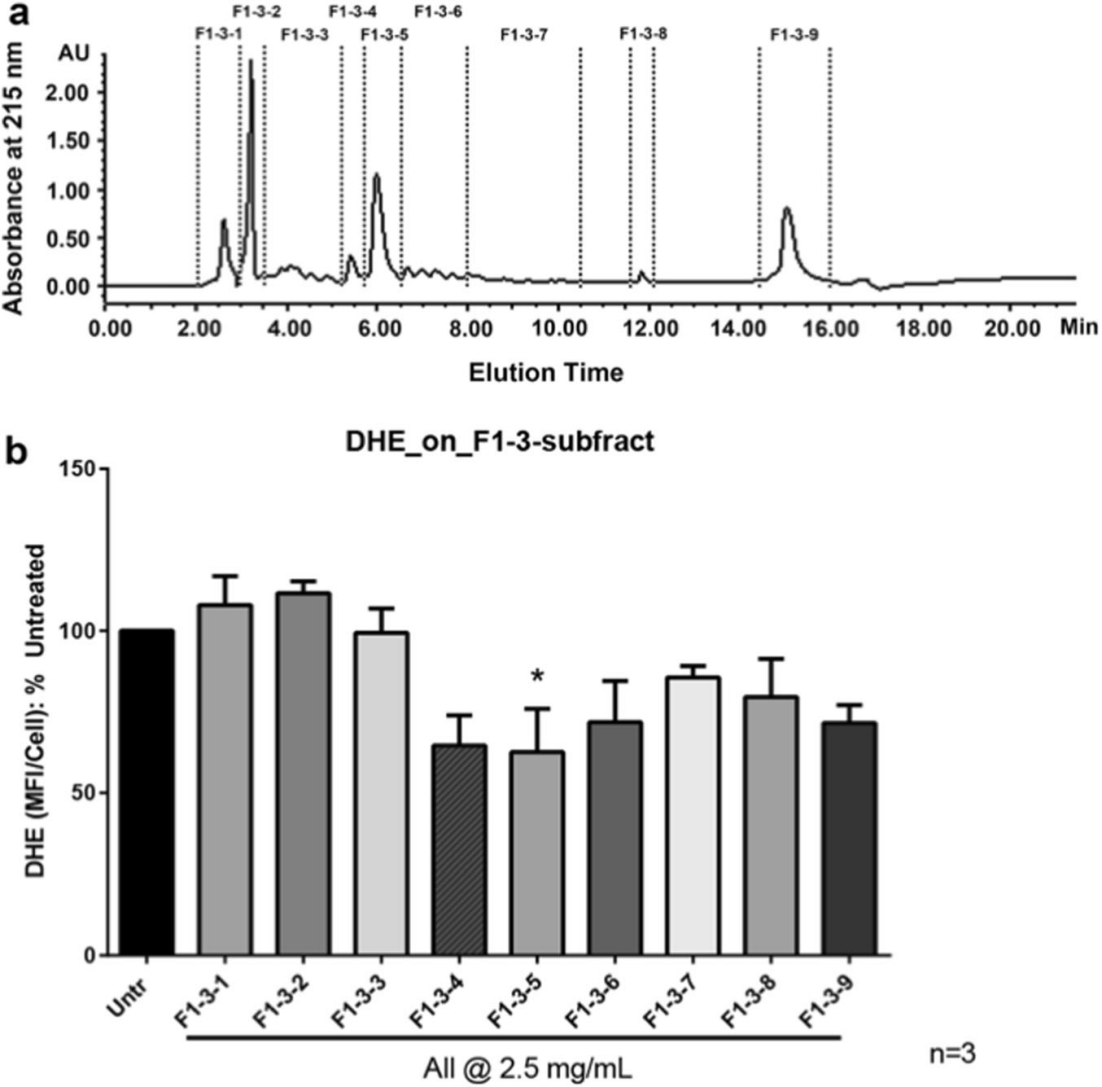
**a** Separation peptide fraction (F1-3) on an X-bridge reverse phase C_18_ column coupled with HPLC system and nine fractions were obtained; **b** antioxidant effect of peptide fractions from HPLC fractionation on A7r5 cells. Untr: control; F1-3-1, F1-3-2, … F1-3-8, and F1-3-9: from F1-3 by HPLC separation. Superoxide generation was expressed as fold change in mean fluorescence intensity per cell (MFI/cell) and presented as percentage (%) of the untreated control. Mean ± SEM of three independent experiments are shown. Asterisk indicates *p* < 0.05 compared to the untreated control

Current study used cell-based assays via dihydroethidium (DHE) staining to evaluate the antioxidant activity of the digests. As shown in Fig. 1, the whole egg digests significantly reduced the superoxide level in VSMCs, confirming egg as a potential antioxidant commodity.^20,27,28^ Among the three fractions, F1 showed a significant higher antioxidant activity (*p* < 0.05) at 2.5 mg/mL compared to the untreated (Fig. 1). In addition, the antioxidant activity of F1 was comparable to the activity of the whole egg digests, indicating the majority of the antioxidant peptides was present in the whole egg digests was eluted with F1. Therefore, F1 was subsequently subjected to a cation exchange chromatography and a total of six fractions were obtained (Fig. 2). Fractions of F1-3 and F1-4 showed stronger antioxidant activity than others and were subjected to further separation by RP-HPLC. Nine fractions were collected from F1-3 via RP-HPLC (Fig. 3a), from which, sub-fractions F1-3-4 and F1-3-5 showed a significantly better antioxidant activity (*p* < 0.05) among all the HPLC frations (Fig. 3b). However, all sub-fractions from F1-4 did not show improvement in antioxidant activity (data not shown). Therefore, F1-3-4 and F1-3-5 were subjected to LC-MS/MS for peptide sequencing.

### Identification of antioxidant peptides

**Fig. 4:**
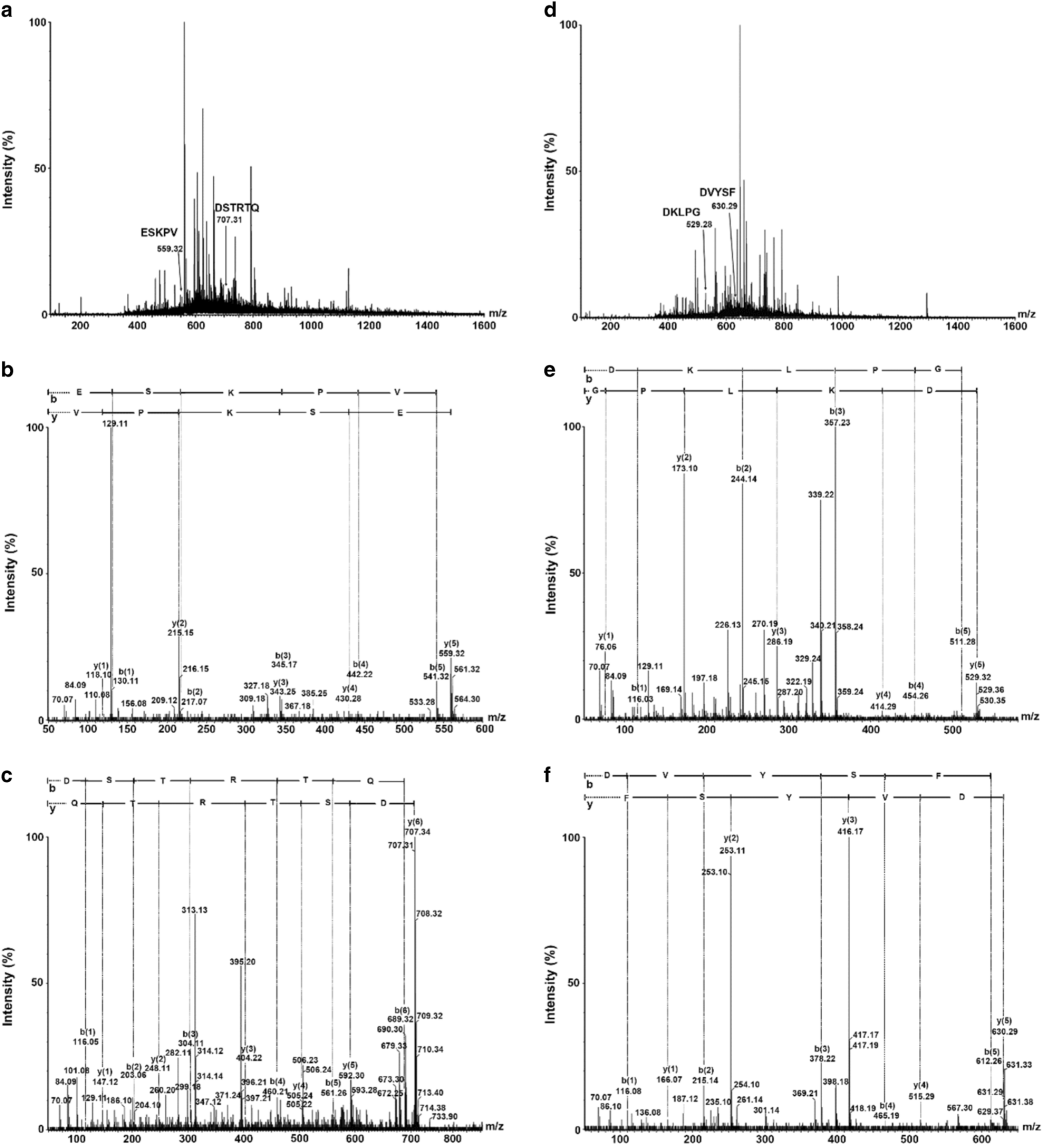
Identification and analysis of antioxidant peptides by LC-MS/MS. Spectra were recorded over the mass/charge (*m*/*z*) ranges of 400–1600 in MS mode **a** and **d**, and 50–1990 (*m*/*z*) in MS/MS mode **b**, **c**, **e**, **f**. **a** MS spectrum of F1-3-4 contains ESKPV and DSTRTQ with molecular weight of 559.32 and 707.31 by single charge; **d** MS spectrum of F1-3-5 contains DKLPG and DVYSF with molecular weight of 529.28 and 630.29 by single charge; **b**, **c**, **e**, **f** MS/MS spectrum of ESKPV, DSTRTQ, DKLPG, and DVYSF for amino acid sequence analysis, respectively

**Fig. 5:**
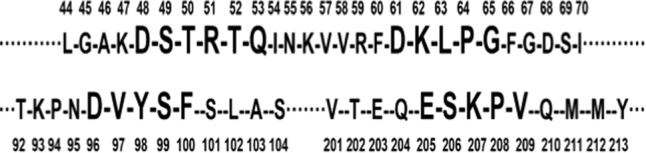
Partial amino acid of ovalbumin (Gallus gallus, gi/212505/gb/AAB59956.1 referring to database of NCBI) from N to C terminal: 48–53 (DSTRTQ), 61–65 (DKLPG), 96–100 (DVYSF), and 205–209 (ESKPV)

Antioxidant fractions were subjected to LC-MS/MS and the results were analyzed by a combination of Mascot searching and MassLynx V4.1 software. Two peptides were identified from F1-3-4 with the amino acid sequence of ESKPV and DSTRTQ, respectively (Fig. 4). The other two peptides with the sequence of DKLPG and DVYSF were identified from fraction F1-3-5 (Fig. 4). All of the four peptides were firstly reported. As indicated by the results from Mascot searching, all four peptides were released form ovalbumin, the most abundant egg white protein, and their position within ovalbumin from N to C terminal: 48-53 (DSTRTQ), 61–65 (DKLPG), 96–100 (DVYSF), and 205–209 (ESKPV) (Fig. 5). Ovalbumin accounts for 54% of egg white proteins,^29^ which makes it highly feasible to generate most peptides from ovalbumin during hydrolysis. Most identified antioxidant peptides were derived from ovalbumin, suggesting ovalbumin is a rich source of antioxidant peptides that can be released by gastrointestinal proteases. However, we could not exclude the feasibility of presence of other antioxidant peptides derived from other egg proteins that have not identified in the study. A series of enzymes were used in TIM-1 system to hydrolyze cooked egg in this study, including pepsin and trypsin. Generally, it is suggested that trypsin preferentially cleaves after amino acids R and K in a peptide chain. Thus, the potential cleaving sites by trypsin in digesting the ovalbumin from whole cooked egg would be between: 47–48, 51–52, 62–63, and 207–208 (Fig. 5). It is also known that presence of P at P1′ position (Peptidecutter), for example 207–208 in Fig. 5, blocks the action of trypsin. Furthermore, if K occurs with either D in position P2 or D in position P1′ (Peptidecutter), such as 47–48 and 61–62 in Fig. 5, the action of trypsin is blocked. Thus, release of these four peptides from ovalbumin was probably not attributed to trypsin digestion. Pepsin preferentially cleaves at F, Y, W, and L in the P1 position or P1′ (Peptidecutter). The potential cleaving sites by pepsin would only be between: 60–61, 62–63, 63–64, 65–66, 97–98, 98–99, and 100–101 (Fig. 5). However, pepsin cleavage is more specific at acidic pH of 1.3, while this specificity is lost at pH ≥ 2 (Peptidecutter). In this study, the pH of TIM system was controlled ≥2, so it was postulated that these four identified peptides were released from ovalbumin by pepsin non-specific cleavage.

### Antioxidant activity validation of the identified peptides

**Fig. 6:**
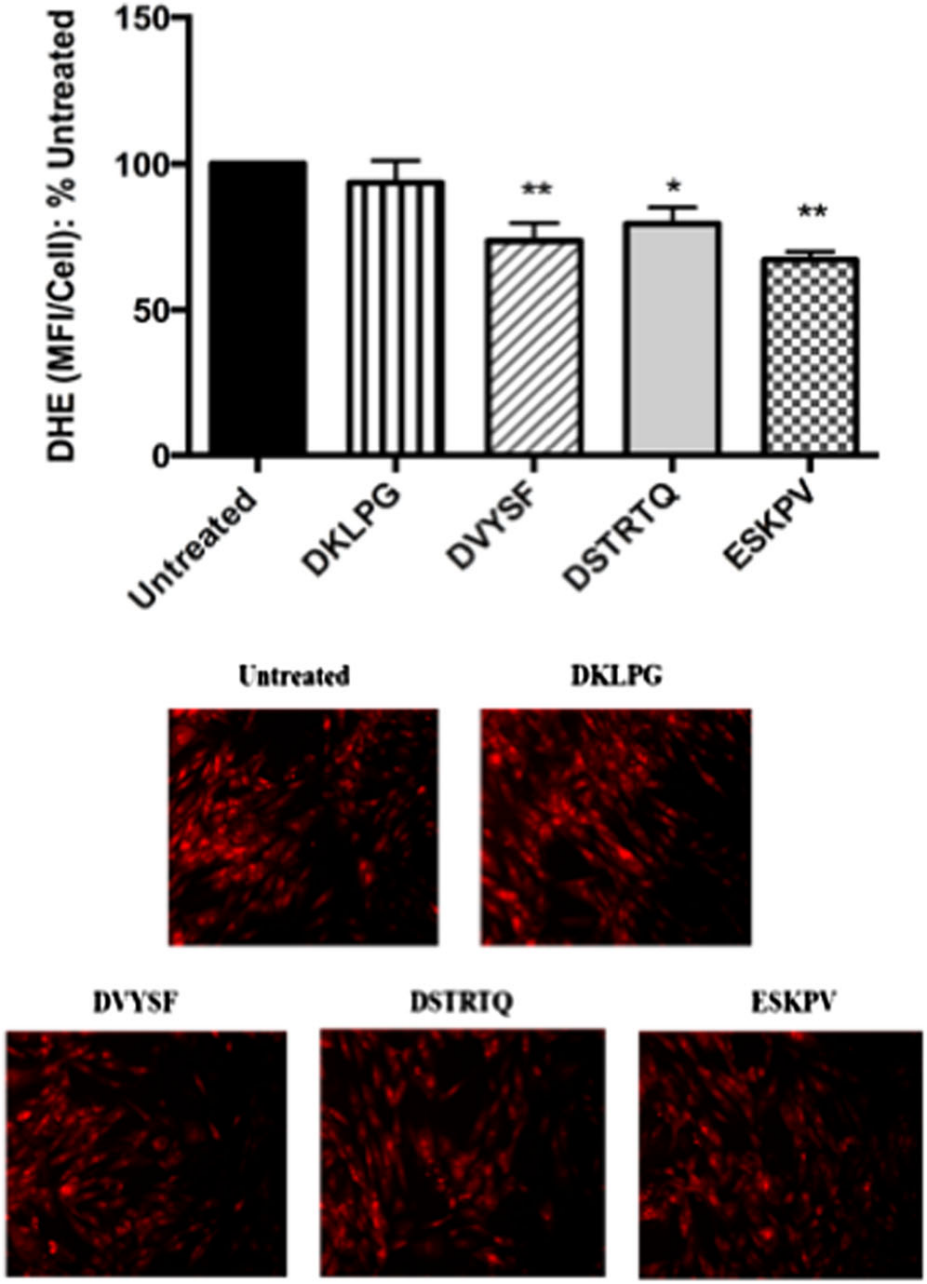
Antioxidant effect of four synthesized peptides on A7r5 cells including DSTRTQ, DKLPG, DVYSF, and ESKPV; as well as Untr: control. The treatment each peptide was 50 μM for 2 h. Superoxide generation was expressed as fold change in mean fluorescence intensity per cell (MFI/cell) and presented as percentage (%) of the untreated control. Mean ± SEM of four independent experiments are shown. Asterisk indicates *p* < 0.05 and double asterisk indicates *p* < 0.01 compared to the untreated control

The above four peptides were then synthesized for activity validation; of which DKLPG did not show antioxidant activity, while ESKPV and DVYSF showed the highest (*p* < 0.05) antioxidant activity (Fig. 6). The peptides identified in this study showed a good antioxidant activity in VSMCs, which is an essential component of vascular wall. The superoxide produced by VSMCs are associated with vascular inflammation, which may eventually lead to vascular remodeling and vascular function abnormality.^30^ The reduced ROS level in VSMCs by the peptide treatments suggested the potential of DSTRTQ, ESKPV, and DVYSF in modulating vascular functions.

Several ovalbumin-derived peptides YAEERYPIL,^11^ AEERYP, DEDTQAMP^14^ were previously reported to have high antioxidant activity. However, these studies were based on chemical antioxidant assays, and the activity of these peptides under a biological system is not known. Previous studies from our lab reported that peptides which showed strong antioxidant activity in chemical assays (ORAC) did not show any activity when tested with human umbilical vein endothelial cells.^21^ In this case, because the chemical assays do not seem to be very reliable and it is important to confirm the activity of a peptide in a more biologically relevant environment, in the current study, we adopted superoxide detection assay on VSMC.

Although the exact mechanism of the activity of these peptides is yet to be confirmed, structural features such as peptide length, amino acid composition, and the position of a single amino acids in the peptide chain can contribute to the bioactivity of a peptide.^31^ Occurrence of N-terminus hydrophobic amino acid (such as A, V, L, I) and presence of aromatic amino acids such as F, Y, W, and also H in the sequence and are known to contribute positively to a peptide’s antioxidant activity.^32^ Aromatic ring as well as the imidazole ring of the H can quench free radicals by donating a proton and maintain the structural stability of the resonance structure. The peptides identified in this study are composed of 5–6 AA residues and from that ESKPV and DVYSF contain V and F in their N-terminus, and also proline and tyrosine in their sequence, respectively, which may contribute to their ability to quench superoxide radicals. The other peptide, DSTRTQ contains glutamine at N-terminus and aspartic acid in the sequence which are reported to play a role in antioxidant peptides.^33^ Overall, structure–activity relationship of antioxidant peptides is not yet clearly understood, but can be greatly affected by the type of terminal amino acids as well as the amino acids in the sequence.

Besides, absorption and bioavailability of DSTRTQ, ESKPV, and DVYSF need to be clarified as well. By now, dipeptides or tripeptides have been identified in human blood after ingestion of protein hydroysate^34,35^; however, knowledge on the absorption and bioavailability of these penta-peptides and hexa-peptides is limited. Although these peptides are likely stable against gastrointestinal enzymes, they may be degraded by epithelial peptidases during absorption; even if they can be absorbed, they are susceptible to further degradation in blood. Therefore, further research is critically important to understand their absorption and bioavailability as well as in vivo antioxidant activity in vivo.

## Conclusions

In this study, antioxidant activity of cooked eggs digested prepared in simulated gastrointestinal system was studied. The egg digests were fractionated on FPLC and RP-HPLC systems and the antioxidant activity was tested on VSMCs. Three ovalbumin-derived peptides were sequenced as DSTRTQ, DVYSF, and ESKPV and their antioxidant activity was confirmed using synthetic peptides. Although cooking of eggs can decrease the antioxidant activity, results from this study suggested that gastrointestinal digestion of cooked eggs generate peptides with more potent antioxidant activity. Overall, this study provides interesting evidence on the formation of beneficial antioxidant peptides after digestion, indicating the potential benefits of egg consumption against oxidative stress in human body.

## Materials and methods

### Materials

Fresh eggs (*n* = 60) were obtained from the Poultry Research Centre of University of Alberta (Edmonton, Canada). Pepsin (from porcine gastric mucosa, 3200 U/mg), trypsin (from bovine pancreas, 7500 U/mg), pancreatin (from porcine pancreas marked with “4XUSP”), and trifluoroacetic acid (TFA) were purchased from Sigma-Aldrich (MO, USA). The electrolyte solutions used for the digestion were designed referring to previous work in our group.^26^ Fresh pig bile was collected from Olymel slaughterhouse (Vallee-Jonction, QC, Canada), and stored at −20 °C until use. HPLC grade acetonitrile (ACN), hexane, ammonium carbonate, and ammonium acetate were purchased from Fisher Scientific (Ottawa, ON, Canada).

### Cooked egg sample preparation

Boiled eggs were prepared as previously reported,^36^ vacuum packed, frozen immediately at −20 °C, and then packed in a Styrofoam box with dry ice for shipment to the Institute of Nutrition and Functional Foods at Laval University (Quebec City, QC, Canada) for digestion.

### Digestion of samples with TIM-1 system

The digestion was carried out using predetermined parameters of TIM-1 modified by Speranza et al.^37^ and also described in our previous work^26^ under subdued light and all solutions/secretions used were purged with nitrogen. The jejunal compartments were connected to specific hollow-filter membranes (MiniKros module M80S-300-01P, Spectrum Laboratories Inc., Rancho Dominguez, CA, USA).^38^ The permeate passing through these membranes was collected and lyophilized.

### Fractionation of digests by C_18_ cartridge

The digest powder was defatted with hexane (the ratio of digest/hexane is 1:5, w/v) for three times, and then desalted by a C18 cartridge column (Sep-Pak^®^ Vac, 35 cc, C18 cartridges, Waters Corporation, Milford, MA, USA), during which the digests were divided into three fractions eluted with the following buffer: Milli-Q water (buffer A), 20% ACN (buffer B), 50% ACN (buffer C), 100% ACN (buffer D). At the same time the eluted fractions were collected and marked as F1, F2, and F3, respectively. These three fractions were then subjected to rotary evaporation (at 45 °C, 720 mm Hg Vaccum) and then lyophilized by freeze dry.

### Separation with FPLC

The fraction with highest activity (F1) was subjected to a cation High-Prep 16/10 column (GE Healthcare Bio-Sciences AB, Uppsala, Sweden) coupled with AKTA explorer 10XT system (GE Healthcare). The column was equilibrated with 3 column volume (CV) of 100% buffer A (10 mM ammonium acetate, pH 4.0 adjusted with acetic acid). The sample was dissolved in buffer A at a concentration of 25 mg/mL, followed by 1 mL of injection. Afterward, fraction was eluted isocratically using the starting buffer for 1.5 CV at a flowrate of 2.5 mL/min, followed by a linear gradient elution to 15% buffer B (0.5 M ammonium carbonate solution at pH 8.8) in 4 CV, to 100% buffer B in 1 CV, and maintain 100% B for 2 CV. The elution was monitored at 215 nm.

### Purification with RP-HPLC

The most active fractions (F1-3 and F1-4) were further purified using RP-HPLC on an X-bridge C18 column (10 × 150 mm, 5 µm, Waters Inc., Milford, MA, USA) coupled with a guard column (10 × 40 mm) on Waters 600 system (Waters, Millford, MA). The buffer A (0.1% TFA in Milli-Q water) and buffer B (0.1% TFA in ACN) were used as the mobile phase. Flow rate was set at 3.0 mL/min and the absorbance was measured at 215 nm. The injection volume is 150 µL at 2 mg/mL sample concentration. The column was equilibrated with 17% buffer B and the sample was eluted by a linear gradient elution from 17 to 25% buffer B in 10 min, to 99% B in 2 min, maintaining at 99% B for 6 min, and then decreasing to 17% B in 2 min and keeping this equilibrating condition for next run.

### Cell culture and antioxidant activity test

A commercially available rat aortic VSMC line, A7r5, was purchased from ATCC (cat# ATCC CRL-1444, Manassas, VA, USA). Cells between passages 3–10 after receiving were used for experiments. Cells were grown in DMEM (Dulbecco’s Modified Eagle Medium) supplemented with 10% FBS and antibiotics (Penicillin-Streptomycin and Gentamicin) at 37 °C with 5% CO_2_ until they reached confluence.

Thus, superoxide generation in VSMC was measured by DHE staining as described previously,^23–25^ and the fluorescence was monitored by a fluorescence microscope (Olympus IX81, Olympus Canada Inc., ON, Canada). Images from three randomly chosen fields were taken for each data point. The number of cells in each field was counted. Mean fluorescence intensity per cell (MFI/cell) was determined based on and total fluorescence intensity divided by the number of cells. Afterwards, fold change was calculated based on MFI/cell and presented as percentage (%) of the untreated control.

### Identification of peptides with LC-MS/MS

Q-TOF premier mass spectrometer (Waters, Milford, MA, USA) coupled with a nano-Acquity UPLC system (Waters, Milford, MA, USA) were used for characterizing peptides from F1-3-4 and F1-3-5. A Waters Atlantis d C18 UPLC column (150 mm × 75 μm, 3 μm, Milford, MA, USA) was applied to separate sample at a flow rate of 3.5 μL/min by using solvent A (0.1% formic acid in Milli-Q water) and solvent B (0.1% formic acid in ACN) as described in our previous work^39^ with slight modification. Briefly, 5 μL of sample was loaded onto a 5 μm trapping column for 5 min using 98% solvent A, followed by a gradient from 98% A to 85% A over 5 min, to 40% A over 35 min, to 5% over 5 min, and maintained at 5% A for 5 min. The flow was ionized by nano-Lockspray in a positive ion mode with capillary voltage of 3.80 kV under 100 °C. The mass/charge (*m*/*z*) within the range of 400–1600 and 50–1990 was recorded in MS mode and MS/MS mode, respectively. The precursor ions within the range of *m*/*z* 3.0 were isolated and a signal threshold of 20 counts/s in total ion current was set to perform auto-MS/MS in the data-dependent acquisition.

Mascot search engine (www.matrixscience.com) was used for database search within NCBI non-redundant (NCBInr) database to match with the parent proteins. MS/MS data was analyzed by MassLynx V4.1 (Micromass UK, Ltd., Wythenshawe, Manchester, UK) combined with manual de novo sequencing.

### Peptides synthesis and activity validation

Four identified peptides with sequences of DSTRTQ, DKLPG, DVYSF, and ESKPV were synthesized by “Gen Script” (Piscatway, NJ, USA) with a purity >98% for antioxidant activity validation. The cells were treated with 50 μM of each peptide for 2 h. Antioxidant activity was determined as described above.

### Statistical analysis

Data are presented as mean ± standard error of mean (SEM) from 3–5 independent experiments. One-way ANOVA with Dunnett’s or Tukey’s post test was to determine the statistical significance. *P-*value < 0.05 was considered to be significant difference.

### Data availability

All data are included in this article.
